# Post-translational Modifications of IκBα: The State of the Art

**DOI:** 10.3389/fcell.2020.574706

**Published:** 2020-11-05

**Authors:** Xiuli Wang, Hanlin Peng, Yaqian Huang, Wei Kong, Qinghua Cui, Junbao Du, Hongfang Jin

**Affiliations:** ^1^Department of Pediatrics, Peking University First Hospital, Beijing, China; ^2^Department of Physiology and Pathophysiology, Peking University Health Science Center, Beijing, China; ^3^Key Laboratory of Molecular Cardiovascular Sciences, Ministry of Education, Beijing, China; ^4^Department of Biomedical Informatics, Centre for Noncoding RNA Medicine, School of Basic Medical Sciences, Peking University, Beijing, China

**Keywords:** IκBα, NF-κB, post-translational modifications, phosphorylation, ubiquitination, SUMOylation

## Abstract

The nuclear factor-kappa B (NF-κB) signaling pathway regulates a variety of biological functions in the body, and its abnormal activation contributes to the pathogenesis of many diseases, such as cardiovascular and respiratory diseases and cancers. Therefore, to ensure physiological homeostasis of body systems, this pathway is strictly regulated by IκBα transcription, IκBα synthesis, and the IκBα-dependent nuclear transport of NF-κB. Particularly, the post-translational modifications of IκBα including phosphorylation, ubiquitination, SUMOylation, glutathionylation and hydroxylation are crucial in the abovementioned regulatory process. Because of the importance of the NF-κB pathway in maintaining body homeostasis, understanding the post-translational modifications of IκBα can not only provide deeper insights into the regulation of NF-κB pathway but also contribute to the development of new drug targets and biomarkers for the diseases.

## Introduction

The nuclear factor-kappa B (NF-κB) is a critical class of transcriptional regulators belonging to the NF-κB/Rel protein family and is found in almost all cell types. In mammals, the NF-κB family has five members, namely RelA (p65), c-Rel, RelB, NF-κB1 (p50) and NF-κB2 (p52), which form homodimers and heterodimers, and the heterodimer of p50 and p65 is the most common form (Giridharan and Srinivasan, 2018). NF-κB plays a pivotal part in the immune response, inflammatory reaction, cell proliferation and apoptosis (Wu et al., 1996; Elewaut et al., 1999; Ellis et al., 2000; Jo et al., 2000). The overactivation of the NF-κB pathway can result in the inflammatory diseases, cardiovascular diseases, autoimmune diseases and cancers; therefore, the NF-κB pathway is tightly regulated to ensure the physiological homeostasis of body systems (Barnes and Karin, 1997; Wong et al., 1999; Kis et al., 2003; DiDonato et al., 2012). The inhibitors of NF-κB (IκBs), including classical IκB proteins (IκBα, IκBβ and IκBε), non-classical IκB proteins (Bcl-3, IκBζ, IκBNS, IκBη and IκBL) and precursor IκB proteins (p105 and p100), are important molecules that regulate its activation. Among these proteins, IκBα is the first in the IκB family to be cloned (Hinz et al., 2012; Annemann et al., 2016) and is one of the members widely studied (Hinz et al., 2012; Annemann et al., 2016). Following exposure to inflammatory cytokines or microbial products, the IκBα protein is degraded, resulting in a decreased inhibitory effect on NF-κB; NF-κB is then translocates from the cytoplasm to the nucleus, where it regulates the transcription of NF-κB target genes (Hoffmann et al., 2006). The regulation of IκBα mainly occurs at post-translational level, including phosphorylation, ubiquitination and SUMOylation (Perkins, 2006). This review focuses on the advances in the molecular structure, function, post-translational modifications and the regulatory mechanisms of IκBα.

## Cellular Localization and Molecular Structure of the IκBα Protein

IκB is an important protein regulating the activity of NF-κB. It forms a trimer with a homologous or heterodimer of NF-κB and retains inactive NF-κB in the cytoplasm (Mothes et al., 2015). IκBα is the most common member of the IκB protein family and can shuttle between the cytoplasm and nucleus. Therefore, it is located in both the cytoplasm and nucleus, and its intracellular distribution is dynamic (Huang et al., 2000).

The molecular mechanism by which IκBα inhibits NF-κB activity is closely related to its protein structure. The human IκBα protein has 317 amino acids (aa) with a molecular weight of 36 kDa. It mainly consists of the N-terminal signal-receiving domain (SRD; 1–72 aa), the intermediate ankyrin repeat domain (ARD; 73–280 aa), the C-terminal proline-glutamic-serine-threonine domain (PEST domain; 281–317 aa) and two nuclear export sequences (NESs; 45–54 aa and 265–277 aa) (Lin et al., 1996; Arenzana-Seisdedos et al., 1997; Johnson et al., 1999; Truhlar et al., 2006; Yazdi et al., 2015).

**FIGURE 1:**
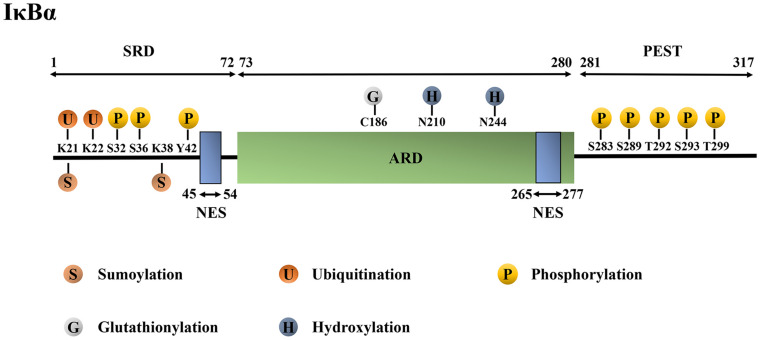
Structure and post-translational modification sites of the human IκBα protein. IκBα, nuclear factor-kappa B inhibitor alpha; SRD, signal-receiving domain; ARD, ankyrin repeat domain; PEST domain, proline–glutamic–serine–threonine domain; NES, nuclear export sequence; K, Lys, lysine; S, Ser, serine; Y, Tyr, tyrosine; C, Cys, cysteine; N, Asn, asparagine; T, Thr, threonine.

The large amount of amide exchange and 8-anilino-1-napthalenesulphonic acid-binding suggest that free IκBα has molten globule structure (Croy et al., 2004). The details are as follows (Figure 1): (1) N-terminal SRD is critical for receiving phosphorylation, ubiquitination and SUMOylation signals as it contains the phosphorylation sites Ser32, Ser36, and Tyr42, ubiquitination sites Lys21 and Lys22, and SUMOylation sites Lys21 and Lys38 (Mabb and Miyamoto, 2007; Hendriks et al., 2017). Additionally, SRD is significant to the activation of NF-κB. Furthermore, it consists of three stable α-helices to lie between residues 8–15 aa, 22–30 aa and 44–50 aa, which complement the six ankyrin repeats (ARs) present in crystallized IκBα. This finding lays a foundation for further studies on the signal transduction of SRD in the IκBα degron (Yazdi et al., 2015). (2) The AR is the characteristic structure of IκB and one of the most common protein sequence motifs that mediates protein–protein interactions (Sedgwick and Smerdon, 1999; Li et al., 2006). Particularly, human IκBα contains six ARs, each consisting of approximately 33 aa. An AR is folded into a β-hairpin, followed by two antiparallel α-helices and a variable loop, which connects it to the next. The α-helical stacks that are thought to form the small hydrophobic cores, whereas the β-hairpin “fingers” form the main protein–protein interaction sites (Sedgwick and Smerdon, 1999; Hendriks et al., 2017). The thermal stability of the ankyrin folding has been suggested to be mainly due to the local interaction between adjacent structural units and needs to be repeated many times to form a stable folding ARD. Amide exchange experiments have revealed that AR3 is the most compact; AR2 and AR4 are less compact; and AR1 and AR6 were solvent-exposed (Croy et al., 2004). ARD contains glutathionylation modification site Cys186 and hydroxylation modification sites Asn210 and Asn244 (Cockman et al., 2006; Kil et al., 2008). (3) The C-terminal PEST domain is highly solvent accessible (Croy et al., 2004); it is not necessary for the binding of IκBα with NF-κB but plays a pivotal role in inhibiting the competitive binding of NF-κB and DNA (Ernst et al., 1995; Sue and Dyson, 2009). The PEST domain contains phosphorylation modification sites Ser283, Ser289, Ser293, Thr291 and Thr299 (McElhinny et al., 1996; Schwarz et al., 1996). (4) Lastly, the two NES suquences are located in the N-terminal SRD domain (45–54 aa) and the C-terminal (265–277 aa). The N-terminal NES is necessary for the shuttling of IκBα between the nucleus and cytoplasm (Huang and Miyamoto, 2001; Lee and Hannink, 2001). Similarly, the C-terminal NES is involved in IκBα-mediated nuclear export of the retroviral oncoprotein v-Rel, which has structural homology with the NF-κB protein family members. However, the substitution of alanine in the C-terminal NES of IκBα did not affect the cytoplasmic relocalization of the mutant IκBα and c-Rel induced by the leptomycin B (Lee and Hannink, 2001). The abovementioned detailed protein structure can be found in references (Croy et al., 2004; Sue and Dyson, 2009; Truhlar et al., 2006; Yazdi et al., 2015).

IκBα and NF-κB form a broad non-continuous binding surface, and all six repeat sequences are involved in the formation of the non-continuous contact surface (Croy et al., 2004). NF-κB family members, such as p65, contain three main domains: the N-terminal domain (NTD), dimerization domain, and transactivation domain (Komives, 2012). AR1–3 contacts the nuclear localization signal (NLS) polypeptide in the N-terminal Rel-homology domain (RHD) of p65, whereas AR4–6 contacts the dimerization domain of p50. Meanwhile, the inner helices of AR5 and AR6 contact the dimerization domain of p65, and the PEST region interacts with the NTD of p65 (Croy et al., 2004). Compared with IκBα in the IκBα–NF-κB complex, free IκBα has higher flexibility (Yazdi et al., 2015). The abovementioned detailed protein structure can be found in references (Croy et al., 2004; Komives, 2012; Yazdi et al., 2015).

A series of conformational changes occur when IκBα binds to NF-κB. First, the ARD of IκBα is slightly twisted, and the distance between residues 166 and 262 increases (Trelle et al., 2016). Thereafter, its AR5 and AR6, whose β-hairpins are highly dynamic in free IκBα, are folded. Amide hydrogen/deuterium (H/D) exchange is useful to identify protein regions that fold upon binding (Truhlar et al., 2006; Trelle et al., 2016). The amide H/D exchange experiments show that the β-hairpins of AR2 and AR3 are significantly resistant to the exchange, whereas those of AR5 and AR6 are completely exchanged in free IκBα within the first minute. When bound with NF-κB, this exchange is significantly reduced. The difference in amide H/D exchange between free and NF-κB-bound IκBα suggests that the β-hairpins in AR5 and AR6 undergo a folding transition upon binding (Truhlar et al., 2006). This folding involves multiple aspects of NF-κB regulation, such as regulating IκBα degradation, mediating its binding to different NF-κB dimers, and possibly promoting the dissociation of NF-κB from DNA (Truhlar et al., 2006). Notably, the AR5 and AR6 of IκBα in the free state are neither random coils nor completely unfolded. In fact, even if the amides in these two repeats are completely exchanged within one minute, when IκBα binds to NF-κB, no new secondary structure is formed (Ferreiro and Komives, 2010). Therefore, the AR5–6 area must be partially folded and may exhibit the molten globular structure in the free state (Sue and Dyson, 2009). Third, the NLS polypeptide of p65 is folded. NMR data revealed that free-form NSL is in a disordered, highly dynamic configuration. After binding to IκBα, its helices 3 and 4 can be folded, with helix 4 being essential for the interaction, particularly the single residue Phe309 of helix 4. The Phe309 ring acts as a “button” fixing the helix 4 to the “buttonhole” formed by Phe77 and Leu80 on the top surface of the ARD of IκBα. This highly specific interaction effectively covers the ARD, resulting in a significant beneficial binding energy (Cervantes et al., 2011). Lastly, the orientation of the p65 NTD is changed. Based on the structure, IκBα uses the bottom of AR6 and C-terminal PEST regions to interact with the p65 NTD. The main interaction occurs between the highly acidic patch of the PEST sequence (i.e., Glu282, Glu284, Asp285, Glu286 and Glu287) and the basic region of the NTD of p65 (Lys28, Arg30, Lys79, Arg158 and His181). Other residues (Trp258, Gln278, Met279 and Pro281) can also stabilize the interface through polar and van der Waals contact. These interactions are suggested to promote the allosteric regulation of DNA binding by NF-κB, i.e., the binding of IκBα changes the direction of the NTD of p65 and locks the NTD into a closed conformation, thereby interfering with the DNA binding of NF-κB (Sue and Dyson, 2009). The abovementioned detailed structure can be found in references (Truhlar et al., 2006; Sue and Dyson, 2009; Ferreiro and Komives, 2010; Cervantes et al., 2011; Trelle et al., 2016).

## Molecular Mechanism of IκBα Regulation of the NF-κB Pathway Activity

**FIGURE 2:**
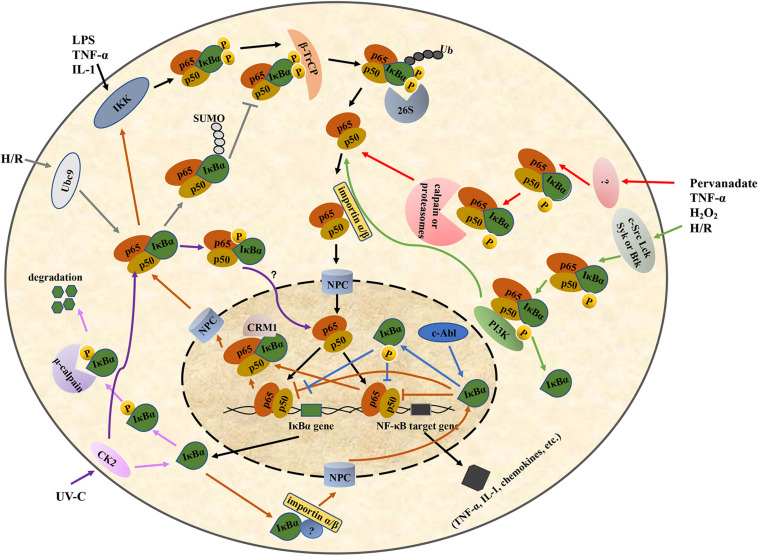
Regulation of the IκBα/NF-κB pathway. IκBα, nuclear factor-kappa B inhibitor alpha; NF-κB, nuclear factor-kappa B. LPS, lipopolysaccharide; TNF-α, tumor necrosis factor-α; IL-1, interleukin-1; H/R, hypoxia/reoxygenation; UV-C, ultraviolet-C; IKK, IκB kinase; CK2, casein kinase II; 26S, 26S proteasome; PI3K, phosphoinositide 3-kinase; Ubc9, ubiquitin-conjugating enzyme 9; β-TrCP, β-transducin repeat-containing protein; NPC, nuclear pore complex; CRM1, chromosome region maintenance 1.

The ARD of IκBα in unstimulated cells binds to the RHD region of the NF-κB dimer, forming a trimer complex in the cytoplasm. When the cells are stimulated, IκBα undergoes post-translational modifications, such as phosphorylation and ubiquitination, and then is degraded by the proteasomes. The NLS in the RHD region of NF-κB is subsequently exposed and recognized by the nuclear transport receptor importin α/β on the cytoplasm. Their binding forms a nuclear complex that is transferred to the nucleus through the nuclear pore complex (NPC) in an energy-dependent manner, thereby initiating the transcription of the target gene (Turpin et al., 1999; Lee and Hannink, 2002) (Figure 2). Because IκBα is also a target gene of NF-κB, IκBα is rapidly replenished when the NF-κB pathway is activated. The newly synthesized IκBα enters the nucleus and translocates NF-κB dimer to the cytoplasm (Turpin et al., 1999; Chen and Greene, 2004) (Figure 2).

IκBα lacks a classical basic NLS, which is a basic amino acid sequence required for nuclear translocation and is originally found in nucleoplasmin and SV40 T antigen. Thus, it cannot enter the nucleus in a classical pathway similar to NF-κB (Turpin et al., 1999). Sachdev et al. found that the AR2 deletion or the mutation in its hydrophobic residues markedly relocalizes IκBα to the cytoplasm (Sachdev et al., 1998). Additional experiments demonstrated that there was a functional discrete NLS in the AR2 region of IκBα, consisting of an N-terminal β-hairpin, an N-terminal α-helix, a C-terminal α-helix, and an adjacent C-terminal β-hairpin (Sachdev et al., 1998). In addition to ARs, some studies have suggested that the nuclear translocation of IκBα requires some unidentified proteins containing a basic NLS and can interact with the ARs of IκBα (Turpin et al., 1999). The nuclear-transport receptor importin α/β heterodimer in the cytoplasm subsequently recognizes the NLS and transports the nascent IκBα into the nucleus (Turpin et al., 1999) (Figure 2).

In the export NF-κB dimer to the cytoplasm, the C-terminal PEST domain and NES of IκBα play an important role. Negatively charged residues in the PEST domain of nuclear IκBα can interact with positively charged residues in the NTD of NF-κB p50, resulting in a conformational change in the NF-κB–DNA complex and facilitating the dissociation of DNA from NF-κB. Thereafter, the PEST domain of IκBα occupies the DNA-binding cavity of NF-κB, with the Glu287 residue interacting with a positively charged residue (typically Arg246) in the p65 NTD, thereby further hindering the binding between DNA and NF-κB (Potoyan et al., 2016). The NF-κB–IκBα complex is specifically recognized by the nuclear export receptor chromosome region maintenance (CRM1)/exportin-1 under the mediation of the NES at the N-terminus of IκBα. It is then transferred to the cytoplasm in the inactive NF-κB–IκBα form via the NPC, thus forming a spontaneous negative-feedback inhibition loop (Sachdev et al., 2000; Figure 2).

Furthermore, studies investigating the regulation of IκBα in the nucleus–cytoplasm shuttle have shown that the masking of the p50 NLS by IκBα in the cytoplasm is transient or incomplete. This leads to the partial exposure of the p50 NLS and causes the NF-κB–IκBα complex to enter the nucleus under the mediation of the NLS. An NES is specifically identified by the nuclear export receptor CRM1, thereby transporting the complex out of the nucleus. Therefore, the subcellular localization of the inactive NF-κB–IκBα complex is not static because of its dynamic shuttling between the nucleus and cytoplasm (Huang et al., 2000).

## Regulation of the Phosphorylation/Dephosphorylation of IκBα

Protein phosphorylation refers to the process of transferring the γ-phosphate group of ATP or GTP to the amino acid residues of serine, threonine or tyrosine of the substrate protein and is catalyzed by protein kinases. “p-” designates that the protein is phosphorylated. A negatively charged phosphate group is added to the amino acid side chain of the protein, resulting in esterification. Esterification changes the protein configuration, activity and its ability to interact with other molecules, thereby greatly affecting cell signal transduction (Hunter, 2000; Kim et al., 2004). Meanwhile, protein dephosphorylation is the reverse process of protein phosphorylation, and is catalyzed by phosphatases (Domingo-Sananes et al., 2011). Therefore, the phosphorylation level of IκBα is regulated by both protein kinase-catalyzed phosphorylation and phosphatase-catalyzed dephosphorylation. Previous studies have shown that three main kinases promote IκBα protein phosphorylation, namely IκB kinase (IKK), casein kinase II (CK2) and tyrosine kinase (Perkins, 2006). The phosphatases that regulate IκBα dephosphorylation include calcineurin and protein tyrosine phosphatase L1 (Pons and Torres-Aleman, 2000; Wang et al., 2018).

### IKK-Dependent Phosphorylation

The IKK complex can be activated by lipopolysaccharides (LPSs), viral proteins, oxygen free radicals, cytokines and other stimuli; it then phosphorylates the Ser36 and Ser32 residues in the SRD region of the N-terminus of IκBα (Traenckner et al., 1995; Hayden and Ghosh, 2004; Kato et al., 2012; Ko et al., 2017; Liu et al., 2018; Safi et al., 2018), leading to ubiquitination and the subsequent degradation of the IκBα in proteasome (Figure 2). IKK-dependent IκBα phosphorylation at the serine residues occurring in the cytosol is a key step in the release of active NF-κB and its nuclear translocation (Yazdi et al., 2017). A molecular dynamics simulation experiment showed that IKK first phosphorylates IκBα at Ser36, changing the local conformation of the N-terminal region and increasing the relative solvent-accessible surface area of Ser32. This enables Ser32 to interact with IKK via phosphorylation (Yazdi et al., 2017).

The subsequent changes in Ser32/Ser36 double-phosphorylated IκBα are as follows. (1) Conformational changes. First, the curved region between p-Ser32 and p-Ser36 is elongated, and the distance between them is increased. Then, the Met1 and Gln3 residues in the N-terminal move away from p-Ser32 to p-Ser36. Finally, the amides in the main chain of Met1 interact with the phosphate group of p-Ser36, whereas those in the main and side chains of Gln3 combine with the backbone carbonyl group of p-Ser36 to form hydrogen bonds (Yazdi et al., 2017). These changes result in the stabilization of the N-terminal tail. (2) Exposure of binding sites. The conformational change caused by double phosphorylation leads to the exposure of Leu34 and Lys21. Leu34 can interact with the β-transducin repeat-containing protein (β-TrCP), the main component of the IκBα-specific E3 ligase, whereas Lys21 is the ubiquitination site of IκBα. (3) Electrostatic interactions. In the non-phosphorylated state, strongly acidic residues (i.e., Asp27, Asp28, and Asp31 upstream of Ser32; and Asp39, Glu40, Glu41, and Glu43 downstream of Ser36) promote the formation of negatively charged regions on or near the protein surface around Ser32/Ser36. In contrast, the negative charge of the phosphate introduced during the double phosphorylation further changes the distribution of the surface charge and forms a more negatively charged protein surface, which is essential for the recognition of IκBα by the β-TrCP–S phase kinase-associated protein 1 (SKP1)–cullin 1 (CUL1)–F-box protein (SCF) complex and the ubiquitination of IκBα (Yazdi et al., 2017). More details can be found in reference (Yazdi et al., 2017).

Moreover, IKK-dependent IκBα phosphorylation might occur in the nucleus and play a transcriptional suppressive effect, together with transcriptional corepressors (Aguilera et al., 2004; Espinosa et al., 2011). Aguilera et al. have found that the nuclear IκBα bound to the promoter of the Notch target gene *hes1* to facilitate the recruitment of histone acetyltransferases and deacetylases, correlating with transcriptional repression in unstimulated cells. In addition, IKK-α and -β were recruited to the *hes1* promoter following TNF-α treatment, correlating with the IκBα phosphorylation, the release of chromatin-associated IκBα and gene transcriptional activation (Aguilera et al., 2004).

Heterozygous gain-of-function mutations associated with Ser32/Ser36 in IκBα can cause autosomal dominant form of anhidrotic ectodermal dysplasia with immunodeficiency. The mutations reported to date include missense mutations of Ser32, Ser36, or adjacent residues and nonsense mutations of upstream residues from Ser32, related to the reinitiation of truncated IκBα translation (Boisson et al., 2017). These mutations inhibit the phosphorylation of both Ser32 and Ser36, as well as the degradation of IκBα, thus enhancing the inhibitory activity of IκBα (Boisson et al., 2017). Fibroblasts, monocytes, and B and T cells of patients with these heterozygous mutations showed abnormal NF-κB regulatory responses to various surface receptor stimuli (Boisson et al., 2017). In addition, all patients suffered from severe B-cell deficiency, whereas some showed specific immunological characteristics, including increased lymphocytes and lack of peripheral lymph nodes. Often, purulent, mycobacterial, fungal and viral infections were also observed (Boisson et al., 2017). In patients with activated B-cell type (ABC) subgroup of diffuse large B-cell lymphoma, p-IκBα level in tissue microarrays of those with poor five-year survival rate was higher than those with good five-year survival rate, suggesting that p-IκBα is an independent prognostic marker of poor survival in ABC patients (Hussain et al., 2013).

### CK2-Dependent Phosphorylation

CK2 is a ubiquitous serine/threonine kinase, and it can phosphorylate multiple serine and threonine residues in the PEST domain of the IκBα protein, including Ser283, Ser289, Ser293, Thr291 and Thr299, among which Ser293 is the most important (McElhinny et al., 1996; Schwarz et al., 1996). When CK2 binds to atypical protein kinase C-zeta (PKC-ζ), Ser293 will be preferentially phosphorylated (Bren et al., 2000).

When IκBα binds to NF-κB, its PEST domain is masked by NF-κB. Therefore, the CK2-dependent phosphorylation of the PEST domain mainly occurs in free IκBα protein, which are degraded directly by the proteasomes in a non-ubiquitin-dependent manner (Mathes et al., 2008). The PEST domain can bind to the μ-calmodulin-like domain of the large subunit of μ-calpain to trigger the degradation of IκBα associated with μ-calpain. The CK2-dependent phosphorylation of the serine/threonine residues in the PEST domain enhances IκBα degradation by μ-calpain (Shumway et al., 1999; Figure 2). CK2 phosphorylation of the free IκBα protein does not depend on stimulation but mainly regulates the basic turnover rate of IκBα in resting cells (Shumway et al., 1999; Mathes et al., 2008). However, some studies have shown that IκBα bound to NF-κB could also undergo CK2-dependent phosphorylation. Particularly, when epithelial HeLa cells are stimulated using short-wavelength ultraviolet radiation, CK2 is phosphorylated and activated by p38 MAP kinase through a phosphorylation- dependent allosteric mechanism, and the activated CK2 then phosphorylates the PEST domain of IκBα, leading to IκBα degradation and NF-κB activation, thus protecting cells from ultraviolet-induced cell death (Sayed et al., 2000; Kato et al., 2003) (Figure 2).

### Tyrosine Kinase-Dependent Phosphorylation

In addition to IKK and CK2, many types of tyrosine kinases are involved in the regulation of IκBα phosphorylation. Here, the modification sites of IκBα depend on the type of tyrosine kinases, stimulation factors and cell types, resulting in similar or opposite effects. The detailed regulation of tyrosine kinase-dependent phosphorylation is as follows.

In the pervanadate-stimulated human myeloid U937 cell, IκBα phosphorylation is induced at the Tyr42 residue, and IκBα is subsequently polyubiquitinated and degraded by the proteasome (Figure 2). This releases the NF-κB and translocates it to the nucleus, activating specific gene expression, including gene expression of IκBα (Mulero et al., 2013a). IκBα degradation and NF-κB activation are observed. However, the detailed regulation of tyrosine kinases in this process is unclear (Mukhopadhyay et al., 2000). U937 is a human leukemic monocyte-lymphoma cell line that is not homogeneous. In HIV-1 permissive clone 10 (plus), NF-κB p65 was constitutively phosphorylated, whereas in non-permissive clone 17 (minus), NF-κB p65 was not constitutively phosphorylated. In addition, both the α1 proteinase inhibitor and LPS induced the phosphorylation of NF-κB p65 Ser536 in the two clones; however, only the dephosphorylation of Ser529 was observed in the plus clone. These results indicated that the phosphorylation/dephosphorylation of NF-κB differs depending on the clone used, suggesting that the IκBα activity is also different (Bristow et al., 2008).

Second, the promotion of IκBα dissociation from the NF-κB complex occurs without its degradation (Figure 2). The tyrosine kinases c-Src, Lck, Syk or Btk are activated by many stimuli, such as TNF-α, pervanadate, hypoxia/reoxygenation, and H_2_O_2_, and phosphorylate IκBα at Tyr42, Tyr289 or Tyr305 residues. For example, when mouse bone marrow macrophages are stimulated by TNF-α, c-Src kinase is activated, and it then phosphorylates IκBα at Tyr42, resulting in the constant c-Src–IκBα association without IκBα degradation. This might be related to the release of the active RelA–p50 from IκBα–RelA–p50 complex and the subsequent of RelA–p50 nuclear translocation because the IκBα/RelA complex remains in the cytosol of c-Src^-/-^ cell treated with TNF-α (Abu-Amer et al., 1998). Meanwhile, when epithelial HeLa cells were stimulated by pervanadate or hypoxia/reoxygenation, c-Src kinase promoted the transcriptional activity of NF-κB by phosphorylating IκBα at Tyr42 (Fan et al., 2003). Pervanadate and hypoxia/reoxygenation can also cause the phosphorylation of IκBα at Tyr42 in Jurkat cells, and this may be mediated by Lck. This activates NF-κB and is associated with the dissociation of IκBα–NF–κB complex (Imbert et al., 1996). Furthermore, H_2_O_2_ can induce the IκBα phosphorylation at tyrosine residues through Syk in human myeloid KBM-5 cells. This promoted the dissociation of the IκBα–NF–κB complex and the phosphorylation and nuclear translocation of p65 but did not affect IκBα degradation (Takada et al., 2003). Similarly, in anti-IgM-stimulated B cell, Btk is rapidly activated; thereafter, it then phosphorylates IκBα at Tyr305 and Tyr289 residues in the cytosol and associates with phosphorylated IκBα, which correlates with the nuclear translocation of p65 and mediated the early transcriptional activation of NF-κB-responsive genes activated via B cell receptor triggering (Pontoriero et al., 2019). Btk and NF-κB are upregulated in acute and chronic lymphocytic leukemia (Pal Singh et al., 2018). In addition, an inactivated mutation in Btk can lead to B-cell immunodeficiency, such as X-linked agammaglobulinemia, in humans (Pal Singh et al., 2018).

Regarding the mechanism by which IκBα phosphorylation at certain residues, such as Tyr42, activates the NF-κB pathway without accompanying IκBα degradation, the dissociation of IκBα from the NF-κB complex is speculated to be induced by other interacting proteins. Béraud et al. found that phosphoinositide 3-kinase (PI3K) is involved in NF-κB activation induced by IκBα phosphorylation at Tyr42, which is located in a consensus sequence of IκBα that binds to the SH2 domain of the PI3K p85 regulatory subunit. When Jurkat T cells were stimulated by pervanadate, the SH2 domain of PI3K p85 regulatory subunit could specifically bind to the phosphorylated Tyr42 of IκBα, causing the dissociation of IκBα from NF-κB/IκBα complex (Béraud et al., 1999).

Lastly, in human embryonic kidney 293T cells and human osteosarcoma U2OS cells, the nuclear non-receptor tyrosine kinase c-Abl can enhance the stability of nuclear IκBα through Tyr305 phosphorylation, allowing IκBα to accumulate in the nucleus and thereby inhibiting NF-κB activation caused by TNF-α stimulation (Kawai et al., 2002).

IκBα phosphorylation at serine and tyrosine residues plays distinct roles in different pathophysiological processes. For instance, in cardiomyopathic mice with cardiac-specific expression of TNF-α (TNF-1.6 mice), NF-κB activation was completely blocked after crossing them with IκBα ^(S32*A, S*36*A, Y*42*F)*^ transgenic mice; however, it only partially blocked after crossing with IκBα ^(S32*A, S*36*A)*^ transgenic mice. In addition, NF-κB activation following acute injury, including TNF-α and ischemia/reperfusion was completely blocked in both IκBα ^(S32*A, S*36*A, Y*42*F)*^ and IκBα ^(S32*A, S*36*A)*^ transgenic mice. These findings suggested that IκBα phosphorylation at tyrosine serves as the second signal for NF-κB activation in the pathogenesis of certain disease, although IκBα phosphorylation at Ser32 and Ser36 is dominant in the regulation of NF-κB activation, thus providing guidance for exploring the multiple targets of IκBα phosphorylation inhibitors in drug design and discovery (Brown et al., 2005).

### Regulation of Dephosphorylation

As described in the abovementioned studies, almost all of IκBα phosphorylation modifications promote NF-κB activation. Similarly, the dephosphorylation of IκBα is critical for inhibiting the activation of NF-κB. Studies have revealed that calcineurin in astrocytes can be activated by insulin-like growth factor-I, which leads to site-specific dephosphorylation of p-Ser32, inhibition of IκBα degradation, and subsequent nuclear translocation of NF-κB p65 stimulated by TNF-α (Pons and Torres-Aleman, 2000). In addition, the protein tyrosine phosphatase L1 inhibits the development of high-grade serous ovarian cancer through dephosphorylating p-Tyr42 to stabilize IκBα and attenuate the nuclear translocation of NF-κB (Wang et al., 2018). Thyme quinone (a natural compound isolated from *Nigella sativa*) can induce the release of superoxide anion and hydrogen peroxide in ABC cells which promoted IκBα dephosphorylation, arrested NF-κB p65 nuclear translocation, and finally inhibited the NF-κB pathway-mediated ABC cell survival pathway (Hussain et al., 2013).

## Regulation of the Ubiquitination/Deubiquitination of IκBα

Ubiquitination is a cascade reaction involving the ubiquitin-proteasome-pathway (UPP), which is important for protein degradation in eukaryotes and is related to many biological processes including the cell cycle, apoptosis and inflammation. IκBα ubiquitination occurs following IκBα phosphorylation at Ser32/Ser36 and is recognized by the proteasome complex. The ubiquitinated IκBα is degraded in the proteasome, accompanied by the release of active NF-κB and the activation of the NF-κB pathway (Kanarek and Ben-Neriah, 2012).

**FIGURE 3:**
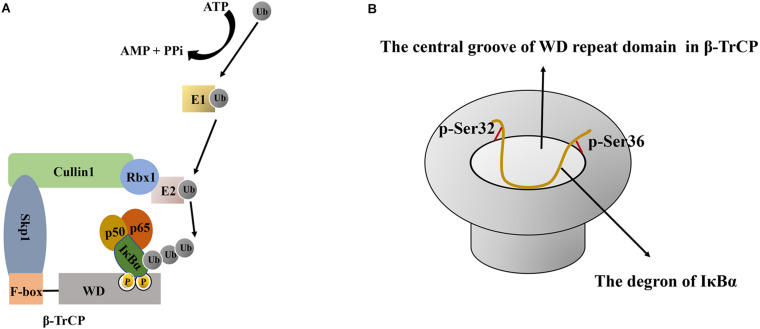
The schematic diagram of IκBα ubiquitination and the interaction between β-TrCP and IκBα. **(A)** Schematic diagram of IκBα ubiquitination. First, E1- activating enzyme activates ubiquitin in an ATP-dependent manner, and then, the E1-ubiquitin intermediate transfers ubiquitin to E2 to form an E2-ubiquitin intermediate. Finally, IκBα ubiquitination requires a specific E3 ubiquitin ligase comprising Skp1, Cullin1, Rbx1, and β-TrCP. As a scaffold protein, Cullin1 can bind to the N-terminus of Skp1 and the C-terminus of Rbx1. Rbx1 binds to E2 and promotes the conversion of ubiquitin from E2 to IκBα, whereas the β-TrCP protein has two characteristic domains, namely the N-terminal F-box domain and the C-terminal WD repeat domain. The F-box domain of β-TrCP is mainly responsible for binding to Skp1, whereas the WD repeat domain is essential for IκBα recognition. **(B)** Schematic diagram of the interaction between the central groove of the WD repeat domain in β-TrCP and the degron of IκBα. All six residues in the IκBα degron are in contact with β-TrCP, and the phosphate groups of p-Ser32 and p-Ser36 bind at the edge of the groove. Ub, Ubiquitin; ATP, adenosine triphosphate; AMP, adenosine monophosphate; PPi, pyrophosphoric acid; IκBα, nuclear factor-kappa B inhibitor alpha; E1, E1 ubiquitin-activating enzyme; E2, E2 ubiquitin-conjugating enzyme; Skp1, S phase kinase-associated protein 1; β-TrCP, β–transducin repeat–containing protein; WD, tryptophan-aspartic acid.

The ubiquitination of IκBα is regulated by E1 ubiquitin-activating enzyme, E2 ubiquitin-conjugating enzyme, and E3 ubiquitin ligase (Kanarek and Ben-Neriah, 2012; Skaar et al., 2013). First, E1 ubiquitin-activating enzyme catalyzes the formation of a thioester bond between the C-terminal glycine residue of ubiquitin and the cysteine residue of E1. This activates ubiquitin in an ATP dependent manner and initiates ubiquitination modification. Thereafter, E1-ubiquitin intermediates transfer ubiquitin to E2, forming an E2-ubiquitin intermediate (Olsen et al., 2010; Singh et al., 2017). Finally, IκBα ubiquitination requires a specific E3 ubiquitin ligase, SCF^β-TrCP^, comprising four subunits: Skp1, Cullin1, Rbx1 and β-TrCP. The crystal structure of SCF^β−TrCP^ shows that Cullin1, as a scaffold protein, can bind to the N-terminus of Skp1 and the C-terminus of cyclin Rbx1. Rbx1 can bind to E2 and promote the switch of ubiquitin from E2 to the substrate (Kanarek et al., 2010) (Figure 3). Meanwhile, the β-TrCP protein consists of two characteristic domains, the N-terminal F-box domain (190–228 aa) and the C-terminal tryptophan-aspartic acid (WD) repeat domain (301–590 aa) (Bai et al., 1996; Deshaies, 1999). The F-box domain was defined because it was first found in cyclin F. Particularly, the F-box domain of β-TrCP is mainly responsible for binding to Skp1, in which Ile143 and Leu152 residues play an important role in maintaining the hydrophobic surface of the β-TrCP–Skp1 interaction (Herter and Fuchs, 2002). The WD repeat domain is critical for substrate recognition (Hu et al., 2017) (Figure 3) and is a typical seven-blade propeller domain with an overall doughnut shaped. Each blade in WD repeat domain contains a conserved WD motif comprising approximately 40 residues and folded into four anti-parallel β chains. The central hole of the WD repeat domain usually mediates interactions with other proteins (Hu et al., 2017). In human IκBα, a degradation sequence, DSGLDS (31–36 aa), has been identified. Upon treatment with stimulating factors (such as TNFα), its Ser32 and Ser36 residues were phosphorylated to induce a conformational change in IκBα that is recognized by the WD repeat domain of the β-TrCP protein (Yaron et al., 1997).

The following changes occur when IκBα combines with β-TrCP: 1) Conformational changes. The 24-residue phosphorylated peptide (24p-IκBα, 21–44 aa) in the free state exhibits a curvature around the ^31^DpSGLDpS^36^ residue. Upon binding with β-TrCP, in addition to the curve corresponding to the ^31^DpSGLDpS^36^ motif, the peptide has two bending regions (Lys22–Asp31 and Met37–Glu43) on both sides (Pons et al., 2007). NMR and crystal structure indicated that the interaction between IκBα and β-TrCP is due to the center curvature. The central bending of the DpSGLDpS motif region is responsible for high-affinity binding, and the N-terminal (^25^LLDDRH^30^) and C-terminal (^35^DpSMKDE^40^) turning regions can enhance the interaction between IκBα and the β-TrCP protein (Pons et al., 2007). 2) Interaction based on charge. In the DDR^29^HDpS^32^GLDpS^36^MKDE^40^E fragment, except for Gly33, Leu34 and Met37, all chain residues form a charged surface, which provides a reasonable binding area with the surface of the charged protein β-TrCP (Pons et al., 2007). 3) Interaction between WD40 and the degradation sequence of IκBα. The central groove through the middle of the WD propeller structure can accommodate the degradation sequence of IκBα. All six residues in the IκBα degradation sequence are in contact with β-TrCP. Particularly, the aspartic acid side chain, the skeleton of the hydrophobic residue glycine and a spacer residue are inserted into the farthest recess, enabling intermolecular contact in mostly buried environments. Meanwhile, the phosphate groups of p-Ser32 and p-Ser36 bind at the edge of the groove and, along with aspartic acid, form the maximum number of contacts through hydrogen bonding and electrostatic interactions with the β-TrCP residues around the groove (Kanarek et al., 2010) (Figure 3). All seven WD repeats of β-TrCP facilitate contact with the substrate, and p-Ser32 may be the major site of interaction with β-TrCP (Yazdi et al., 2017). The abovementioned detailed protein structural information can be found in references (Pons et al., 2007; Kanarek et al., 2010). The recognition of IκBα by β-TrCP prompts the transfer of ubiquitin from the thioester intermediate formed with E2 to Lys21 and Lys22 of IκBα. When the first ubiquitin molecule is attached to the target protein, the other ubiquitin molecules are successively linked to the Lys48 residue of the ubiquitin molecule linked to the substrate in the presence of SCF^β-TrCP^ E3, thereby forming a polyubiquitin chain, which is a target signal for the recognition and degradation of IκBα by the proteasome (Figure 2). Ubiquitinated IκBα enters the 26S proteasome and is degraded in the 20S catalytic center. Ubiquitins can be hydrolyzed from IκBα by deubiquitinating enzymes, such as ubiquitin-specific protease for reutilization, which is the deubiquitination of IκBα (Rape et al., 2006). In summary, the affinity of SCF^β-TrCP^ and the activity of deubiquitinating enzymes co-regulate IκBα ubiquitination.

In addition to β-TrCP, the muscular atrophy F-box protein has been shown to ubiquitinate IκBα in cardiomyocytes (Usui et al., 2011). Additionally, a novel E3 ligase, F-box and WD repeat domain-containing protein 7 (FBW7), targeting IκBα has been identified; its upregulation promotes the ubiquitin-dependent IκBα degradation, NF-κB activation, and the subsequent intestinal inflammation caused by intestinal epithelial cells, whereas its inhibition has the opposite effects (Meng et al., 2020).

## Sumoylation of IκBα

Small ubiquitin-related modifier (SUMO) is a class of peptides comprising 98 aa and has low sequence identity with ubiquitin but forms a 3D structure similar to that of ubiquitin. To date, four members of the SUMO family (SUMO1–4) have been identified in mammalian cells (Carbia-Nagashima et al., 2007). SUMO proteins are expressed in a precursor or inactive form with a short C-terminus. Sentrin/SUMO-specific proteases (SENPs) possess hydrolase activity and cleave the inactive precursor of SUMO at the C-terminus to generate the active or mature SUMO. The human SENP family is composed of SENP1, SENP2, SENP3, SENP5, SENP6, SENP7, and SENP8. However, SENP8 had no effect on SUMO, and only SENP1, SENP2 and SENP5 can proteolyze the precursor SUMO proteins (Yeh, 2009; Kumar and Zhang, 2015; Kunz et al., 2018). SUMOylation is the process of protein coupling with SUMO through three enzymatic steps similar to the ubiquitin-binding cascade reaction.

The SUMOylation of IκBα can be activated by hypoxia/reoxygenation, adenosine receptor agonists (Liu et al., 2009). Particularly, in mammalian cells, the SUMO-specific E1-activating enzyme is the heterodimer SAE1/SAE2, and the E2-binding enzyme is the ubiquitin-conjugating enzyme 9 (Ubc9). In the presence of ATP, the IκBα SUMOylation *in vitro* only requires SAE1/SAE2 and Ubc9 and not E3 protein ligase. However, whether the E3 protein ligase is necessary for the SUMOylation of IκBα *in vivo* remains unclear (Desterro et al., 1999).

**FIGURE 4:**
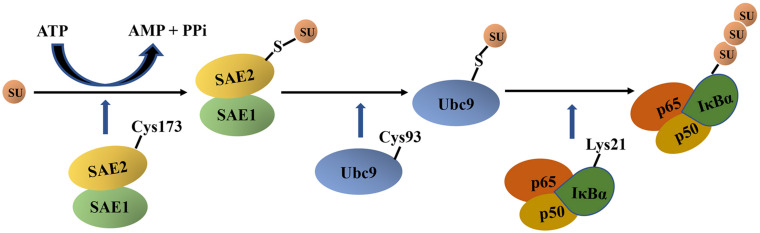
The schematic diagram of IκBα SUMOylation. First, a C-terminal glycine residue of mature SUMO forms a thioester bond with the active site Cys173 in SAE2 in an ATP-dependent manner. Thereafter, SUMO is transferred to the catalytic cysteine residue Cys93 of Ubc9, and Ubc9 directly interacts with IκBα and transfers SUMO to IκBα. Next, the glycine residue at the C terminal of mature SUMO forms a covalent isopeptide bond with the Lys21 side chain in IκBα. IκBα, nuclear factor-kappa B inhibitor alpha; SU, SUMO, small ubiquitin-like modifier; SAE, SUMO-1–activating enzyme; Ubc9, ubiquitin-conjugating enzyme 9; ATP, adenosine triphosphate; AMP, adenosine monophosphate; PPi, pyrophosphoric acid.

IκBα SUMOylation based on *in vitro* experiments is described as follows (Figure 4). First, the active site Cys173 in SAE2 forms a thioester bond with the glycine residue at the C-terminus of SUMO in an ATP-dependent mannerr. This transfers SUMO to the catalytic cysteine residue Cys93 of Ubc9 (Olsen et al., 2010). Thereafter, Ubc9 directly interacts with IκBα by recognizing the consensus sequence ψKxE (ψ represents a hydrophobic amino acid residue, whereas x represents any amino acid residue) and transfers SUMO to IκBα. The glycine residue in the C-terminus of SUMO forms a covalent isopeptide bond with the side chain of Lys21 in IκBα (Rodriguez et al., 2001). In addition, the results of high-throughput screening for SUMOylated proteins recently identified Lys38 as another SUMOylation site of IκBα (Hendriks et al., 2017).

Complex interactions between the SUMOylated IκBα and other IκBα modifications, such as ubiquitination and phosphorylation, affect the functional effect of IκBα SUMOylation (Scherer et al., 1995; Desterro et al., 1998; Hay et al., 1999; Liu et al., 2009; Aillet et al., 2012; Mulero et al., 2013b; Marruecos et al., 2020). The effects of the complex interactions is described as follows. (1) The SUMO1 modification of IκBα inhibits the ubiquitination of Lys21 and Lys22 in IκBα and blocks IκBα degradation (Figure 2). Studies have shown that the SUMO1 overexpression blocks the ubiquitination and degradation of IκBα caused by TNF-α and IL-1 in African green monkey kidney fibroblast cell line COS7 (Desterro et al., 1998). The following mechanisms may be involved in the inhibition of IκBα ubiquitination by SUMO1 modification. First, because Lys21 is the site of both SUMO and ubiquitin modifications, SUMO1 can competitively bind to it. Many SUMO1 modifications can stabilize IκBα and protect it from degradation by the proteasome system, thus inhibiting NF-κB activation (Desterro et al., 1998). Moreover, the NTD of IκBα is flexible, and the SUMOylation of Lys21 may cause the conformational change of IκBα to prevent it from being recognized by the SCF complex. Second, ubiquitination can occur solely at Lys22 to promote the proteasome-mediated degradation of IκBα without modification at Lys21. However, the SUMOylation of Lys21 having a spherical structure may prevent Lys22 ubiquitination by the β-TrCP SCF complex (Scherer et al., 1995; Mabb and Miyamoto, 2007). (2) The SUMO2/3 modification of IκBα can promote the ubiquitin-dependent degradation of IκBα (Aillet et al., 2012). Studies have shown that the SUMO2/3 modification of IκBα can be detected under physiological conditions. Upon TNF-α stimulation, the heterologous chains of SUMO2/3 and ubiquitin can promote IκBα degradation by 26S proteasome more effectively than SUMO2/3 or ubiquitin alone (Aillet et al., 2012). (3) The SUMOylation of IκBα is blocked by its phosphorylation. Phosphorylated IκBα at Ser32/Ser36 cannot undergo SUMOylation, suggesting that the SUMO modification is only effective before IKK phosphorylates IκBα (Hay et al., 1999). (4) The phosphorylation and SUMOylation of IκBα (PS-IκBα) coexist and exert a NF-κB independent transcription repressive effect (Mulero et al., 2013b; Marruecos et al., 2020). In unstimulated basal keratinocytes, IκBα undergoes IKK-independent phosphorylation modification and is then modified by SUMO2/3 to form PS-IκBα, which is retained in the nucleus. Nuclear PS-IκBα binds to the chromatin of the regulatory regions of target genes, such as *HOX* and *IRX*, and inhibits their transcription by recruiting the polycomb repressive complex 2 (PRC2), which is involved in the regulation of skin homeostasis and development. When keratinocytes are exposed to inflammatory stimuli or differentiation inducer, PS-IκBα dissociates from the chromatin, resulting in the inability to recruit PRC2 to the promoters of the target genes and subsequently upregulating gene expression. This ultimately establishes a link between inflammatory signals and skin homeostasis (Mulero et al., 2013b). Similarly, in intestinal crypt cells, phosphorylated and SUMOylated nuclear IκBα bind the PRC2 subunit SUZ12 and suppress the expression of fetal intestinal stem cell genes, which participated in the regulation of intestinal stem cell homeostasis and the intestinal response to inflammation, damage and repair (Marruecos et al., 2020). The abovementioned mechanism underlying nuclear PS-IκBα deepens the understanding of the IκBα function (Mulero et al., 2013b; Marruecos et al., 2020).

In addition to acting as a hydrolase to promote SUMO maturation, SENP possesses isopeptidase activity and cleaves the isopeptide bond between the glycine at the C-terminal of SUMO and lysine of the substrate protein. This leads to the release of the SUMO protein from its substrate, thereby arresting SUMOylation (Kumar and Zhang, 2015). SENP1, SENP2, SENP3, SENP5, SENP6, and SENP7 have been revealed to possess isopeptidase activities. SENP1 and SENP2 have broad specificity for SUMO-1/2/3, whereas the other SENPs preferentially dissociate SUMO-2/3 from its substrate (Kumar and Zhang, 2015).

## Glutathionylation of IκBα

Glutathione is a tripeptide composed of glutamic acid, cysteine and glycine. It exists in an oxidized (GSSG) form and a reduced (GSH) form, which can protect cells from damage by reactive oxygen species and heavy metals (Lv et al., 2019). Glutathionylation is a reversible post-translational modification involving formation of a disulfide bond between glutathione and protein cysteine thiol and can be reversed by glutathionase (thiol transferase) in a process called for de-glutathionylation.

In IκBα, Cys186 is the target of glutathionylation (Kil et al., 2008). The stimulation of HeLα cells with an oxidant (such as diamide) that induces GSH oxidation can activate IκBα glutathionylation (Kil et al., 2008). Compared with the native protein, glutathionylated IκBα displayed increased quantum yield of the emission spectra and a red shift of the maximum emission wavelength at 337 nm. Moreover, the accessibility of the hydrophobic regions in glutathionylated IκBα is higher than that in the native protein (Kil et al., 2008). Regarding the functional effect of glutathionylated IκBα, Kil et al. found that the glutathionylation of IκBα suppressed IKK- and CK2-dependment IκBα phosphorylation and IκBα ubiquitination *in vitro*. The mechanism may be related to the conformational change of IκBα induced by glutathionylation (Kil et al., 2008). In addition, Seidel et al. found that IκBα glutathionylation inhibited IκBα degradation, NF-κB p65 nuclear entry, and NF-κB/DNA binding, therefore downregulating the expression of NF-κB target genes in airway smooth muscle cells (Seidel et al., 2011).

## Hydroxylation of IκBα

Both *in vitro* and *in vivo* experiments have demonstrated that human hypoxia-inducible factor hydroxylase can effectively hydroxylate Asn244 and Asn210 in IκBα, with Asn244 being more effectively hydroxylated than Asn210. Both these residues are located in the hairpin loops that connect the ARs (Cockman et al., 2006). However, the regulatory effect of IκBα hydroxylation modification on the NF-κB signaling pathway remains unclear. Considering that the hydroxylation of collagen prolyl and lysyl residues can promote the stability of the extracellular matrix structure, asparagine hydroxylation in IκBα is speculated to play a similar role in stabilizing the protein structure (Myllyharju and Kivirikko, 2004).

## Therapeutic Opportunities Based on IκBα Modulation

Abnormal NF-κB activation is related to various diseases, such as cardiovascular, neurodegenerative and autoimmune diseases and cancer. For example, the increase in p-IκBα and the activation of NF-κB p65 were detected in monocrotaline-induced pulmonary hypertensive rats, mice with Alzheimer’s disease, rats with autoimmune myocarditis, and gastric cancer caused by *Helicobacter pylori* (Nozaki et al., 2005; Feng et al., 2017; Zhang et al., 2017; Fang et al., 2019). As a key regulator of the NF-κB signaling pathway, IκBα has attracted increasing research attention and its post-translational modification provides new therapeutic opportunities for diseases related to abnormal NF-κB activation (Vrábel et al., 2019).

For instance, proteasome inhibitors have been clinically used in the treatment of multiple myeloma (MM) (Tundo et al., 2020). Previous studies have demonstrated that the dysregulation of NF-κB pathway contribute to the development and clinical manifestations of MM via NF-κB target genes, including the growth factor interleukin-6 and insulin-like growth factor-1, cell cycle regulators cyclin D and c-Myc, and pro-angiogensis factors vascular endothelial growth factor-C and placental growth factor (Vrábel et al., 2019). Because the ubiquitination and subsequent degradation of IκBα in the proteasome are predominant mechanisms of the NF-κB pathway activation (Feng et al., 2017; Tundo et al., 2020), bortezomib, a reversible proteasome inhibitor, was used in the treatment of MM. Bortezomib binds directly to the β5-subunit of the proteasome and inhibits the enzymatic activity of the proteasome complex. As reported by many clinical trials, bortezomib alone or in combination with other drugs has dramatically improved the outcomes for patients with relapsed MM (Richardson et al., 2003; Lonial et al., 2011), which was in accordance with experimental results showing that bortezomib inhibited NF-κB activation in MM cells by blocking IκBα degradation in the proteasome (Hideshima et al., 2002). In addition, carfilzomib, a second-generation proteasome inhibitor, has been shown to significantly reduce mortality compared with bortezomib. Carfilzomib is the first drug to increase the overall survival rate of MM patients (Dimopoulos et al., 2017). Thereafter, the first oral proteasome inhibitor, ixazomib, was developed for the treatment of relapsed or refractory MM (Narayanan et al., 2020). Compared with bortezomib, ixazomib inhibited tumor proteasome activity for a longer duration and exhibited greater antitumor effect in OCI-Ly10 and PHTX22L lymphoma mouse models (Narayanan et al., 2020). In addition to carfilzomib and ixazomib, second-generation proteasome inhibitors include oprzomib, dilanzomib and malizomib, and their clinical trials are underway (Tundo et al., 2020).

It should be emphasized that in addition to the NF-κB pathway, proteasome inhibitors regulate many other important cellular pathways that depend on proteasome function and affect these signal transduction pathways in both normal and cancer cells, resulting in some limitations to their clinical use, including dose-limiting side effects and the rapid onset of secondary drug resistance (Begalli et al., 2017). For example, bortezomib usually causes peripheral neuropathy, whereas carfilzomib can cause cardiotoxicity, acute renal failure, pulmonary toxicity and other adverse reactions (Narayanan et al., 2020). Therefore, it seems reasonable that the inhibition of IκBα ubiquitination may represent a safer alternative to proteasomal inhibition for blocking NF-κB activation.

Furthermore, because of their central role in regulating IκBα ubiquitination, β-TrCP and its interaction with IκBα are attractive targets (Kanarek and Ben-Neriah, 2012). Studies have found that brain-specific TRIpartite motif protein 9, A49, and GS143 can inhibit IκBα ubiquitination and NF-κB activation by binding to β-TrCP (Nakajima et al., 2008; Mansur et al., 2013; Shi et al., 2014). The IκBα-super repressor (IκBα-SR), a mutant form of IκBα, which can neither be phosphorylated nor ubiquitinated, is found to inhibit NF-κB activation. Furthermore, the intraperitoneal injection of purified IκBα-SR-loaded exosomes attenuates mortality and systemic inflammation in septic mouse models (Choi et al., 2020). IκBα-SR can also increase the sensitivity of pancreatic cancer and pancreatic ductal adenocarcinoma to chemotherapeutics and inhibit tumor development in hepatitis-related cancer murine models (Sato et al., 2003; Pikarsky et al., 2004; Waters et al., 2015). Moreover, the microinjection of IκBα phosphopeptides into TNF-α-stimulated cells blocked NF-κB activation by competing with endogenous IκBα for binding to β-TrCP (Yaron et al., 1997). Cyclic IκBα phosphopeptides, the backbone of which was cyclized to improve stability and selectivity, were designed to effectively block IκBα ubiquitination (Qvit et al., 2009). However, because the E3 ubiquitin ligase SCF^β-TrCP^ has many substrates other than IκBα, potential adverse effects of β-TrCP inhibitors due to the accumulation of SCF^β-TrCP^ may occur, thus partly limiting their clinical usage. Therefore, further in-depth studies are required to develop clinical drugs targeting IκBα post-translational modification.

Moreover, a new method, fragment-based virtual E-pharmacophore screening, has been developed to promote the development of drug design targeting on the NF-κB/IκBα complex (Kanan et al., 2019). An excellent study conducted by Kanan et al. investigated the structure of the binding pocket of NF-κB p65/p50 heterodimer complex with IκBα in detail, and therefore constructed the e-pharmacophore models to discover potential ligands with strong binding affinity as candidate NF-κB/IκBα inhibitors. Furthermore, the screening small molecules and known inhibitors were deeply analyzed on the MetaCore/MetaDrug platform and their therapeutic activity, pharmacokinetic and toxicity profile were predicted. The abovementioned computational biological study might provide a novel approach for exploring the potent and low toxicity NF-κB/IκBα inhibitors and give a new perspective of clinical treatment of diseases with abnormal NF-κB activation.

## Conclusion and Perspectives

In this review, we summarized the recent literature on post-translational modification of IκBα, including the associated structural changes and regulatory processes and their functional effect on NF-κB activation. These studies provide a theoretical basis for therapeutic interventions targeting IκBα post-translational modification for NF-κB-related disease. Many clinical trials on drugs targeting the IκBα post-translational modification achieved significant clinical benefit, thus confirming the therapeutic significance of these post-translational modifications.

However, there are many notable issues in the drug development based on the post-translational modification of IκBα to be addressed for their future clinical usage. First, considering the unavoidable adverse effects of the IκBα-related NF-κB inhibitors (Pancheri et al., 2020), the pathophysiological mechanisms and the significance of the IκBα–NF-κB pathway in the development of diseases should be further clarified to provide the best ratio of benefit to risk. Second, the drug release controllability to ensure the controllable release rate under certain specific pathophysiological conditions should be taken into account because of the vast regulatory role of NF-κB pathway (Chen and Stark, 2019). For example, endogenous sulfur dioxide (SO_2_) is found to play extensive regulatory roles in the cardiovascular system as a novel gasotransmitter (Huang et al., 2016). Along with the increasing studies on the pathophysiological significance of endogenous SO_2_ in the cardiovascular diseases, SO_2_ donors and prodrugs with different triggering mechanisms, such as thiol-activated SO_2_ prodrugs, thermally activated SO_2_ prodrugs and hydrolysis-based SO_2_ prodrugs, were designed in recent years (Day et al., 2016; Wang and Wang, 2018). The abovementioned studies might provide useful evidence for the drug design targeting the inhibition of NF-κB pathway. Third, novel post-translational modifications, such as persulfidation/sulfhydration (Mustafa et al., 2009; Fu et al., 2019), are being discovered. As reported in the previous studies, persulfidation of target protein characterized by a chemical modification of protein cysteinyl thiols to persulfides could change the protein function (Du et al., 2014). Therefore, whether this new kind of post-translational modification occurs in the IκBα protein should be investigated. Fourth, the interaction among different post-translational modifications of IκBα is important and should be stressed. For example, the discovery of PS-IκBα in the nucleus provides a new target for the treatment of intestinal inflammatory diseases and skin cancers such as squamous cell carcinoma (Mulero et al., 2013b; Colomer et al., 2017; Marruecos et al., 2020).

Finally, the localization of NF-κB and IκBα in the mitochondrion and their regulation of mitochondrial DNA activities should be investigated. NF-κB p65 and IκBα have been detected in the mitochondrial (Bottero et al., 2001; Zamora et al., 2004; Pazarentzos et al., 2014). In addition, TNF-α stimulation induces mitochondrial IκBα phosphorylation and proteasome-independent IκBα degradation and promotes the accumulation of mitochondrial NF-κB p65, thus leading to decreased expression of mitochondrial DNA cytochrome c oxidase III and cytochrome b in U937 cells (Cogswell et al., 2003). Further, in the retina of dark-adapted rats exposed to bright light, the mitochondrial translocation of NF-κB p65 was associated with decreased cytochrome c oxidase III expression (Tomita et al., 2016). Studies on the mitochondrial IκBα–NF-κB may provide an alternative mechanism by which IκBα–NF-κB pathway regulates mitochondrial homeostasis, thus serving as a novel target for the treatment of mitochondria-related diseases.

## Author Contributions

XW sorted out, reviewed, and analyzed the literatures, drew the diagrams, and wrote the manuscript. HJ devised the concept. HP sorted out, reviewed, and analyzed the literatures and revised the manuscript. HJ, WK, QC, JD, and YH supervised the writing. All the authors revised and approved the final version of the manuscript.

## Conflict of Interest

The authors declare that the research was conducted in the absence of any commercial or financial relationships that could be construed as a potential conflict of interest.

## Abbreviations

β -TrCP: β -transducin repeat-containing protein
ABC: activated B-cell type
AR: ankyrin repeat
ARD: ankyrin repeat domain
CK2: casein kinase II
Cox III: cytochrome c oxidase III
CRM1: chromosome region maintenance 1
FBW7: F-box and WD repeat domain-containing protein 7
GSH: glutathione
GSSG: glutathione disulfide
HKII: hexokinase II
I κ B α-SR: I κ B α-super repressor
I κ B: inhibitor of NF- κ B
IKK: I κ B kinase
LPS: lipopolysaccharide
MM: multiple myeloma
NES: nuclear export sequence
NF- κ B: nuclear factor-kappa B
NLS: nuclear localization signal
NPC: nuclear pore complex
NTD: N-terminal domain
PEST: proline–glutamic–serine–threonine
PI3K: phosphoinositide 3-kinase
PKC- ζ: protein kinase C-zeta
PRC2: polycomb repressive complex 2
PS-I κ B α: phosphorylation and SUMOylation of I κ B α
RHD: Rel-homology domain
SAE: SUMO-1-activating enzyme
SCF: S phase kinase-associated protein 1 (SKP1)–cullin 1 (CUL1)–F-box protein
SENP: sentrin/SUMO-specific protease
SO_2_: sulfur dioxide
SRD: signal-receiving domain
SUMO: small ubiquitin-like modifier
Ub: ubiquitin
Ubc9: ubiquitin-conjugating enzyme 9
UPP: ubiquitin-proteasome-pathway
WD: tryptophan-aspartic acid.
